# Multiseptated Cystic Mass in the Pelvic Cavity: A Case Report of a Leiomyoma with Cystic Degeneration Mimicking a Peritoneal Inclusion Cyst

**DOI:** 10.2174/0115734056502596260825214914

**Published:** 2026-09-11

**Authors:** Jung Hoon Oh, Weon Jang

**Affiliations:** 1 Biomedical Research Institute of Jeonbuk National University Hospital, Jeonju, South Korea;; 2 Department of Radiology, Jeonbuk National University Medical School, Jeonju, South Korea

**Keywords:** Leiomyoma, Cystic degeneration, Peritoneal inclusion cyst, Differential diagnosis, Magnetic resonance imaging, Bridging vessel sign

## Abstract

**Background:**

Uterine leiomyomas are common benign neoplasms, but atypical presentations such as extensive cystic degeneration are rare and can pose significant diagnostic challenges. When pedunculated and predominantly cystic, these tumors can closely mimic other pelvic pathologies, particularly Peritoneal Inclusion Cysts (PIC).

**Case Presentation:**

A 23-year-old nulliparous woman presented with lower abdominal pain. Computed Tomography (CT) and Magnetic Resonance Imaging (MRI) revealed a large multiseptated cystic mass in the pelvic cavity, located posterolaterally to the uterus. An adjacent smaller cystic mass demonstrated T1 hyperintensity and T2 shading on MRI, suggestive of an endometrioma. Due to the multiseptated appearance and the presence of underlying endometriosis as a predisposing factor, the initial radiologic diagnosis was a PIC with an associated endometrioma. The patient subsequently underwent laparoscopic surgery, which revealed a large pedunculated subserosal leiomyoma arising from the uterine fundus with extensive cystic degeneration. Coronal MRI demonstrated a connecting stalk observed intraoperatively, and histopathology confirmed the diagnosis of leiomyoma with cystic degeneration and associated endometriosis.

**Conclusion:**

Uterine leiomyoma with cystic degeneration should be considered in the differential diagnosis of multiseptated cystic pelvic masses, especially when ovaries are identified separately. Careful assessment using multiplanar reconstruction to identify a connecting stalk or the bridging vessel sign is essential to avoid diagnostic pitfalls and ensure appropriate surgical planning.

## 1 INTRODUCTION

Uterine leiomyomas, commonly referred to as fibroids, are the most prevalent benign solid neoplasms of the female pelvis, affecting up to 70–80% of women of reproductive age. These tumors originate from the smooth muscle cells of the myometrium and are highly dependent on female hormones, particularly estrogen and progesterone, for their growth. While a significant proportion of leiomyomas remain asymptomatic and are discovered incidentally during routine pelvic examinations or imaging, when symptomatic, they can frequently lead to clinical symptoms such as abnormal uterine bleeding, pelvic pain, or mass-related effects [1, 2].

As these tumors outgrow their vascular supply, they undergo various histopathological changes known as degeneration. Hyaline degeneration is the most common form, but more complex patterns such as red, myxoid, and cystic degeneration also occur. In cases of degeneration in giant leiomyomas, hyaline degeneration usually evolves into a large cystic cavity, potentially simulating other causes of uterine and adnexal masses [3, 4].

The imaging diagnosis of typical leiomyomas is characterized by well-circumscribed, homogeneous masses with iso to mildly hypointense signal on T1-weighted MRI and characteristically low signal intensity on T2-weighted MRI [2]. However, atypical presentations, specifically those involving extensive multiseptated cystic degeneration, pose a significant diagnostic challenge. Such masses can closely mimic other pelvic pathologies, including complex ovarian malignancies or Peritoneal Inclusion Cysts (PIC), especially when the fibroid is pedunculated or lacks a clear connection to the uterus. PIC, which result from fluid entrapment by peritoneal adhesions, often share the multiseptated cystic appearance seen in advanced leiomyoma degeneration [3, 5].

In this report, we present a case of a large uterine leiomyoma with atypical multiseptated cystic degeneration that mimicked a PIC. This article aims to discuss the diagnostic pitfalls encountered during workup and highlight key imaging clues necessary to distinguish degenerated leiomyomas from their cystic mimics.

## 2 CASE REPORT

A 23-year-old nulliparous woman presented to our gynecologic clinic with a 1-week history of lower abdominal pain. Initially, the pain was intermittent and colicky, and the patient presumed it to be dysmenorrhea because it began shortly after her menstruation. However, the pain persisted, progressively worsened, and radiated to the right flank 1 day before presentation, prompting her visit to our clinic. Her gynecologic history was notable for menarche at 11 years of age, regular 28-day menstrual periods lasting 7 days, and menorrhagia. She had no prior surgical history. On presentation, physical examination revealed tenderness in both lower quadrants. Initial laboratory tests, including tumor markers, were within normal limits (CA19-9: 16.77 IU/mL, CA-125: 20.4 IU/mL).

Contrast-enhanced Computed Tomography (CT), which was performed on the day of presentation, demonstrated a well-defined, multiseptated cystic mass measuring approximately 10.8 × 7.7 × 8.6 cm in the pelvic cavity, located posterolaterally to the right side of the uterus. Within the cystic mass, there was another relatively hyperattenuating cystic mass (Fig. **1A**). The right ovary was adjacent to the mass and clearly delineated (Fig. **1B, C**). For further characterization, pelvic MRI was performed subsequently 6 days after the CT scan. The large cystic mass showed homogeneous high signal intensity on T2-weighted imaging with multiple thin internal septations and no significant enhancement after contrast administration (Fig. **2A**). Within the cyst, another well-defined cystic mass with high signal intensity on T1-weighted imaging and T2 shading was identified, suggestive of an endometrioma (Fig. **2B, C**). Neither mass showed diffusion restriction on Diffusion-Weighted Imaging (DWI), suggesting the absence of highly cellular tissue components and making entities such as aggressive neoplasms or abscesses less likely. Based on these findings, the radiologic impression was a PIC with an intracystic endometrioma. Other differential diagnoses of a multiloculated cystic pelvic mass also include ovarian cystadenoma, hydrosalpinx, pelvic abscess, and lymphatic cysts. Ovarian cystadenomas typically arise from the ovary and may demonstrate enhancing septa or mural nodules. Hydrosalpinx often shows a tubular configuration with incomplete septa, whereas pelvic abscesses are usually associated with inflammatory symptoms and diffusion restriction. Lymphatic cysts may appear as multiloculated cystic masses but generally lack a clear relationship to the uterus. In the present case, however, the multiseptated cystic appearance and the coexistence of endometriosis led to the initial impression of a PIC.

Following consultation with gynecologic surgeons, laparoscopic excision was performed 3 weeks after the initial presentation. The surgery was scheduled because of the patient's persistent symptoms, the large size of the mass, and diagnostic uncertainty regarding its origin. Intraoperatively, a large pedunculated subserosal mass with a marked cystic component was identified arising from the right fundal region of the uterus and connected by a distinct stalk, confirming its uterine origin (Fig. **3**). The adjacent smaller cystic mass corresponded to an endometriotic cyst. The larger mass contained clear mucoid fluid, while the smaller mass contained dark, chocolate-colored fluid. Histopathologic examination confirmed uterine leiomyoma with extensive cystic degeneration and associated endometriosis (Fig. **4A-C**).

The postoperative course was uneventful. Following surgery, her abdominal pain and menorrhagia significantly improved. During the follow-up visit, pelvic ultrasonography demonstrated no evidence of complications or recurrence. Given her young age, nulliparous status, and the potential risk of diminished ovarian reserve following endometrioma cystectomy, serum anti-Müllerian hormone levels were serially monitored, and postoperative hormonal therapy was initiated to reduce the risk of recurrence.

## 3 DISCUSSION

Leiomyomas are the most common uterine neoplasms. Cystic degeneration is an uncommon form of leiomyoma degeneration, occurring in approximately 4% of cases. It is considered to be an extreme sequela of edema or hyaline degeneration where the hyaline material liquefies. In young nulliparous women, the risk of developing fibroids is reportedly higher than in parous women, potentially due to continuous ovarian steroid exposure without pregnancy-related hormonal rest periods [6]. This uninterrupted hormonal stimulation can accelerate tumor growth, outpacing the blood supply and thus promoting acute ischemic central liquefaction within the fibroid [3, 4]. Large and small cystic spaces develop in the edematous, acellular center, which appear as round, well-demarcated areas with fluid signal intensity that is low on T1-weighted imaging and high on T2-weighted imaging without enhancement. When pedunculated, a degenerated leiomyoma can be difficult to differentiate from adnexal and pelvic cystic masses [5, 7].

In this case, the pelvic mass demonstrated no restricted diffusion on DWI, indicating a lack of highly cellular tumorous components or purulent material. Thus, the DWI findings supported a benign cystic process, although they did not permit reliable differentiation among peritoneal inclusion cyst, endometrioma, and cystically degenerated leiomyoma.

Among the differential diagnoses, PICs typically result from fluid accumulation trapped by peritoneal adhesions, most commonly following prior surgery, pelvic inflammatory disease, or endometriosis. Characteristic imaging features include a multiseptated, thin-walled cystic mass with an irregular configuration that conforms to the contours of surrounding pelvic structures [8]. The spider-in-a-web appearance, caused by the entrapment of a morphologically normal ovary within the septations, is a key diagnostic clue for PIC [9, 10]. The initial impression favored a peritoneal inclusion cyst despite the absence of the classic “spider-in-a-web” sign because of the mass’s typical multiseptated cystic appearance and the relative rarity of extensive cystic degeneration in leiomyomas. In addition, endometriosis is a well-recognized predisposing factor for peritoneal inclusion cysts, and the presence of a coexisting endometrioma further reinforced this interpretation. Previous studies have reported a close association between peritoneal inclusion cysts and endometriosis. Takeuchi *et al*. reported that all six patients with postoperative peritoneal cysts had concomitant endometriosis, suggesting a pathogenic role of endometriosis in PIC formation. Furthermore, Stumpf *et al*. described a PIC associated with an entrapped ovary and an endometriotic cyst, creating a diagnostic dilemma. In contrast, our mass represented extensive cystic degeneration of a leiomyoma that mimicked a PIC despite the coexistence of endometriosis/endometrioma [11, 12].

Meanwhile, other radiologic findings can be used complementarily to determine the uterine origin of a pelvic mass, providing further diagnostic evidence. For instance, the bridging vessel sign is a typical MRI or US finding indicating blood vessels originating from the uterus supplying the pelvic mass. On MRI, it appears as enhancing tubular structures on contrast-enhanced T1-weighted imaging or as flow voids on T2-weighted imaging. On Doppler US, the vessels are shown as signals flowing from the uterus to the pelvic mass [13]. In our case, however, such findings were not clearly identified, which posed a diagnostic challenge in determining the origin of the mass preoperatively. Specifically, the absence of a recognizable bridging vessel sign in the present case may be attributable to both tumor-related and technical factors. Extensive cystic degeneration resulted in near-complete replacement of the solid leiomyomatous component, potentially reducing the visibility of feeding vessels that typically originate from the uterus. In addition, the mass was connected to the uterus by a relatively small stalk, which may have contained only a limited vascular pedicle. The large size of the cystic component may also have compressed or displaced adjacent vessels, further obscuring their visualization. From a technical perspective, routine imaging planes may not have been optimal for depicting small bridging vessels, particularly when the uterine attachment is subtle. These factors likely contributed to the absence of a definite bridging vessel sign on preoperative imaging.

Although a comprehensive imaging evaluation was performed initially, identifying the uterine origin of the mass posed a significant diagnostic challenge due to its large size and extensive cystic degeneration. The predominance of fluid components obscured the residual leiomyomatous tissue and stalk, while the mass occupied much of the pelvis and distorted adjacent anatomy. Furthermore, the feeding vessels were likely compressed and obscured by the extensive cystic component. As a result, subtle clues such as myometrial continuity and the bridging vessel sign were virtually unrecognizable on the initial interpretation. Notably, the connecting stalk was not overtly definitive; rather, a stalk-like structure with low T2 signal intensity adjacent to the right fundal region of the uterus was visualized exclusively on coronal images, which was subsequently correlated with the operative findings (Fig. **5A-C**).

Several cases of cystically degenerated leiomyomas mimicking adnexal or ovarian masses have previously been reported. However, the present case differs from previously described cases in several respects. First, the mass demonstrated extensive multiseptated cystic degeneration with only a minimal residual solid component, making recognition of its uterine origin particularly difficult. Second, the coexistence of endometriosis-associated endometrioma strongly favored the diagnosis of a peritoneal inclusion cyst because endometriosis is a well-recognized predisposing factor for PIC formation. Third, neither a definite bridging vessel sign nor an obvious connecting stalk was readily apparent on the initial image interpretation. These factors collectively contributed to a misleading radiologic impression and illustrate an important diagnostic pitfall in pelvic imaging [14, 15]. Awareness of this diagnostic pitfall is essential, as careful assessment of the relationship between the mass, uterus, and ovaries can improve diagnostic accuracy. To facilitate this assessment in clinical practice, we propose a practical diagnostic checklist (Table **1**).

## CONCLUSION

The study reports a rare case of uterine leiomyoma with extensive cystic degeneration presenting as a multiseptated cystic mass that closely mimicked a PIC. When a multiseptated pelvic mass is present, and both ovaries are identified separately, clinicians and radiologists should maintain a high suspicion for uterine-origin masses. Careful evaluation for a potential connecting stalk using multiplanar reconstruction and the bridging vascular sign is essential for establishing the correct diagnosis and distinguishing the mass from its cystic mimics. This approach is crucial for determining the optimal surgical strategy and avoiding diagnostic pitfalls.

## Figures and Tables

**Fig. (1):**
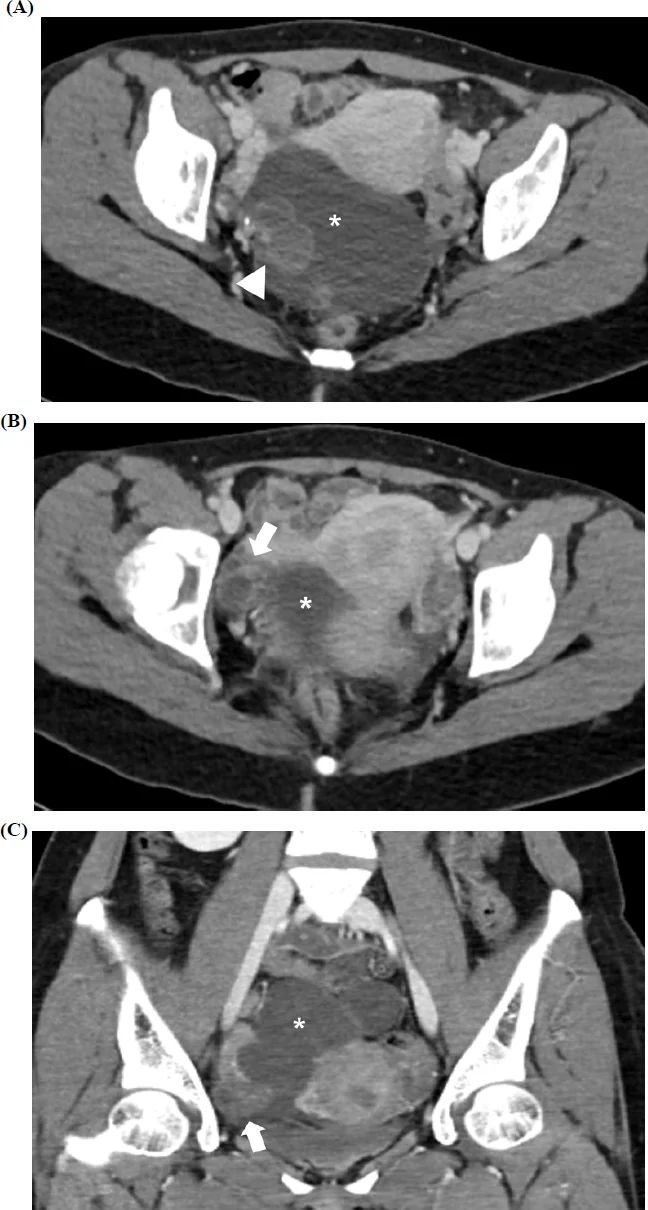
Computed tomography (CT) images. (**A**) A well-defined, multiseptated cystic mass (asterisk) in the pelvic cavity, posterolateral to the uterus on an axial CT image. A relatively hyperattenuating cystic mass (arrowhead) within the mass (asterisk) on an axial CT image. (**B**, **C**) The right ovary (arrows) is clearly visualized and separate from the cystic mass on axial and coronal CT images.

**Fig. (2):**
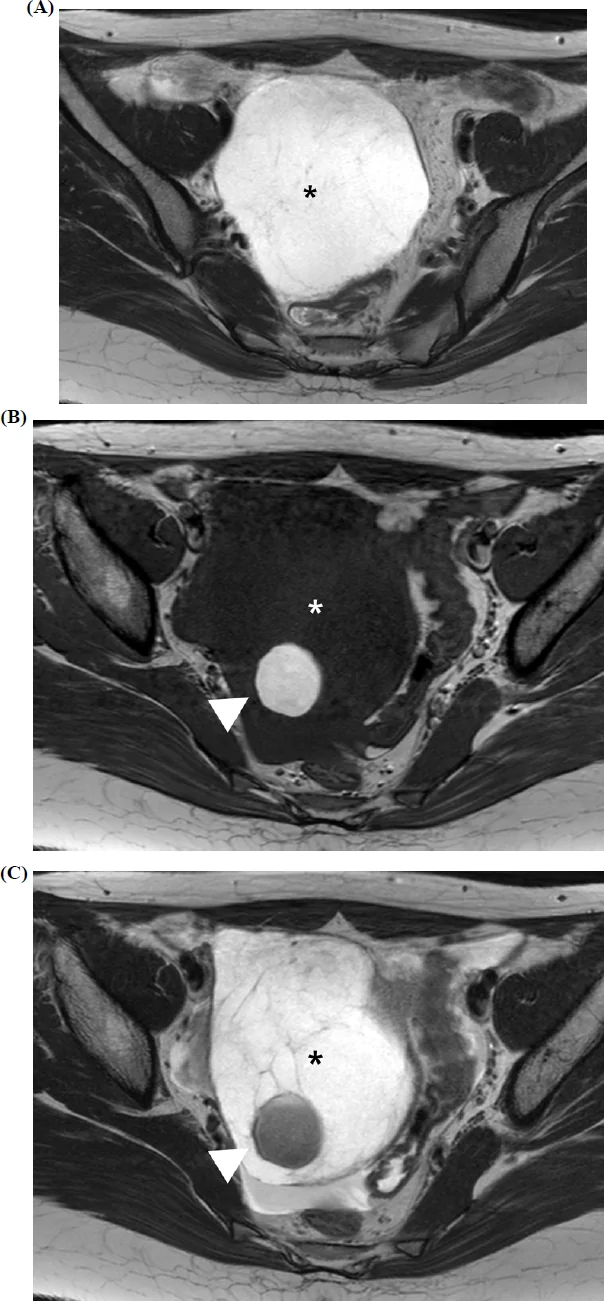
Magnetic resonance imaging (MRI) images. (**A**) T2-weighted image shows a large cystic mass (asterisk) with homogeneous high signal intensity and multiple thin internal septations. (**B**, **C**) A cystic mass (arrowhead) within the mass (asterisk) shows high signal intensity on the T1-weighted image (B) and T2 shading on the T2-weighted image (C), compatible with an endometrioma.

**Fig. (3):**
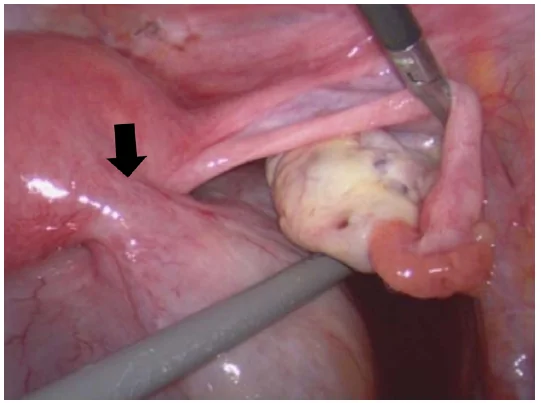
Intraoperative laparoscopic images. An intraoperative laparoscopic image shows a large pedunculated subserosal mass with extensive cystic degeneration arising from the right uterine fundus and connected to the uterus by a distinct stalk.

**Fig. (4):**
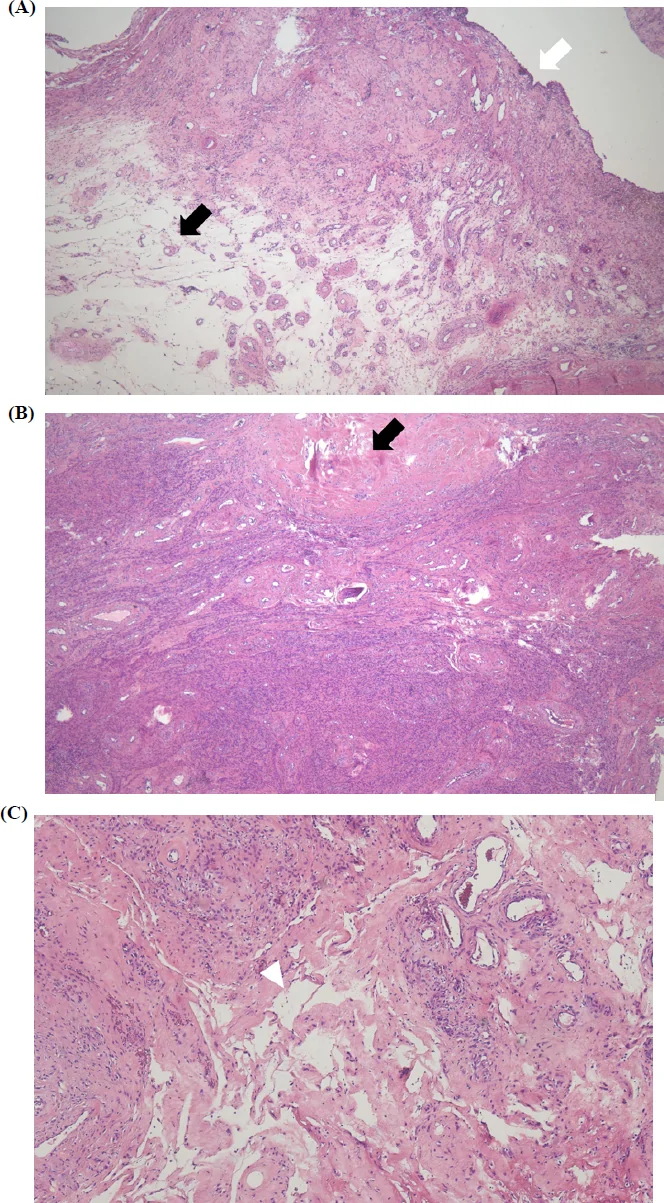
Histopathologic findings. (**A**) Histopathological features of coexisting leiomyoma with secondary degeneration and endometriosis. A representative photomicrograph shows the close anatomical proximity between the leiomyoma lesion and endometriosis. The leiomyoma area exhibits characteristic secondary degenerative changes (black arrow). Adjacent to it, the endometriotic lesion is characterized by endometrial glands and stroma (white arrow) (H&E stain, ×100). (**B**) Microscopic findings of the leiomyoma with cellular areas and focal degeneration. The tumor is predominantly composed of hypercellular areas with interlacing bundles of smooth muscle cells. Mild degenerative changes (arrow) are focally observed in the upper portion of the field (H&E stain, x100). (**C**) Histopathological view of degeneration in leiomyoma. Representative photomicrograph demonstrating detailed features of secondary degeneration inside the tumor. The interlacing smooth muscle bundles are replaced by loose, edematous tissue (arrowhead) representing focal degenerative change. Thickened vascular structures are noted nearby (H&E stain, ×200).

**Fig. (5):**
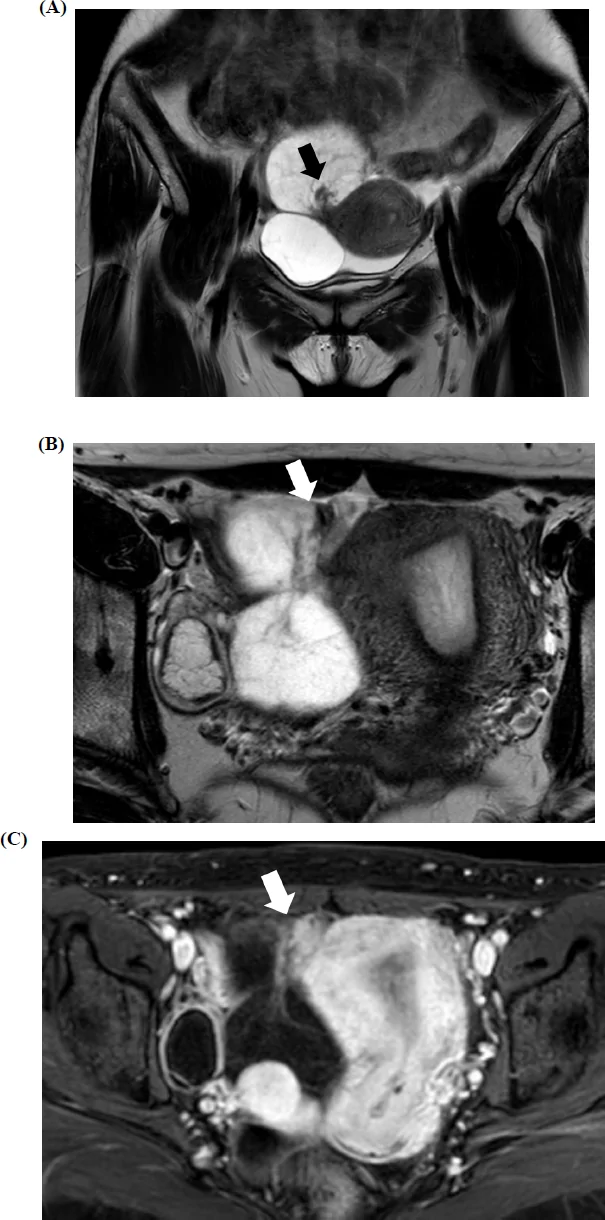
Coronal MRI images. (**A**–**C**) Coronal T2-weighted images show a low-signal-intensity stalk-like structure (arrows) connecting the mass to the right uterine fundus, corresponding to the stalk identified intraoperatively.

**Table 1:** Diagnostic checklist for evaluating multiseptated cystic pelvic masses: distinguishing uterine-origin cystic mass from peritoneal inclusion cyst.

**Clinical Question**	**Favors Uterine Origin**	**Favors PIC / Other Etiology**
**Step 1. Ovarian Status** Are both ovaries identified separately from the mass?	Both ovaries clearly identified separately from the mass.	Ovary entrapped within septations (“spider-in-a-web” sign).
**Step 2. Uterine Connection** Is a connecting stalk or myometrial continuity present?	Low T2-signal stalk or broad-based myometrial continuity identified on multiplanar imaging.	No myometrial continuity or connecting stalk identified.
**Step 3. Bridging Vessel Sign** Is the bridging vessel sign present?	Bridging vessels originating from the uterus identified on CE-T1WI, T2WI flow voids, or Doppler US.	No bridging vessels identified; however, absence alone does not exclude uterine origin.
**Step 4. Clinical Context** Does the clinical context support a specific diagnosis?	Reproductive age / premenopausal patients with no prior pelvic events.	History of pelvic surgery, PID, or endometriosis.

**Abbreviations:** PIC, peritoneal inclusion cyst; PID, pelvic inflammatory disease; CE-T1WI, contrast-enhanced T1-weighted imaging; T2WI, T2-weighted imaging; US, ultrasonography.

## Data Availability

The data and supporting information are available within the article.

## LIST OF ABBREVIATIONS

PIC: Peritoneal Inclusion Cyst
PID: Pelvic Inflammatory Disease
CA-125: Cancer Antigen 125
CA 19-9: Cancer Antigen 19-9
CT: Computed Tomography
MRI: Magnetic Resonance Imaging
T1WI: T1-Weighted Imaging
T2WI: T2-Weighted Imaging
CE-T1WI: Contrast-enhanced T1-Weighted Imaging
DWI: Diffusion-Weighted Imaging
US: Ultrasonography
H&E: Hematoxylin and Eosin

## STANDARDS OF REPORTING

This case report was prepared in accordance with the CARE guidelines.
